# Supplementary material for: Association of tumor necrosis factor-α-308G/A polymorphism with the risk of obstructive sleep apnea: A meta-analysis of 14 case-control studies
Source: PLoS One. 2023 Aug 18;18(8):e0290239. doi: 10.1371/journal.pone.0290239 (PMC10437904; doi:10.1371/journal.pone.0290239)
Supplement: S1 Table — (DOCX) [file pone.0290239.s001.docx]

| **Section and Topic** | **Item #** | **Checklist item** | **Location where item is reported** |
| --- | --- | --- | --- |
| **TITLE** | | |  |
| Title | 1 | The report is identified as a meta-analysis. | 1 |
| **ABSTRACT** | | |  |
| Abstract | 2 | The abstract contains Objective, Methods, Results and Conclusion. | 2 |
| **INTRODUCTION** | | |  |
| Rationale | 3 | Described in the Introduction. | 3-4 |
| Objectives | 4 | Stated in the Introduction. | 4 |
| **METHODS** | | |  |
| Eligibility criteria | 5 | Explained in the Methods. | 5 |
| Information sources | 6 | Specified in the Methods. | 5 |
| Search strategy | 7 | Presented in the Methods. | 5 |
| Selection process | 8 | Specified in the Methods. | 5, Fig 1 |
| Data collection process | 9 | Specified in the Methods. | 5-6 |
| Data items | 10a | Listed and defined in the Methods. | 5-6, Table 1 |
|  | 10b | Listed and defined in the Methods. | 5-6, Table 1 |
| Study risk of bias assessment | 11 | Specified in the Methods. | 5-6 |
| Effect measures | 12 | Specified in the Methods. | 6 |
| Synthesis methods | 13a | Described in the Methods. | 5 |
|  | 13b | Described in the Methods. | 6 |
|  | 13c | Described in the Methods. | 6 |
|  | 13d | Described in the Methods. | 6 |
|  | 13e | Described in the Methods. | 6 |
|  | 13f | Described in the Methods. | 6 |
| Reporting bias assessment | 14 | Described in the Methods. | 7 |
| Certainty assessment | 15 | Described in the Methods. | 6 |
| **RESULTS** | | |  |
| Study selection | 16a | Described in the Results. | 7 |
|  | 16b | Cited in the Results. | 7 |
| Study characteristics | 17 | Cited in the Results. | 7-8, Table 1 |
| Risk of bias in studies | 18 | Presented in the Results. | 12 |
| Results of individual studies | 19 | Presented in the Results, and also presented as Table 2 and Figure2. | 10-11 |
| Results of syntheses | 20a | Summarized in the Results. | 12 |
|  | 20b | Presented in the Results, and also described in the Table 2. | 11 |
|  | 20c | Presented in the Results. | 12 |
|  | 20d | Presented in the Results. | 12 |
| Reporting biases | 21 | Presented in the Results. | 12 |
| Certainty of evidence | 22 | Presented in the Results, and also described in the Table 2. | 7 |
| **DISCUSSION** | | |  |
| Discussion | 23a | Interpreted in the Discussion. | 13-15 |
|  | 23b | Discussed in the Discussion. | 15 |
|  | 23c | Discussed in the Discussion. | 15 |
|  | 23d | Discussed in the Discussion. | 15-16 |
| **OTHER INFORMATION** | | |  |
| Registration and protocol | 24a | The review was not registered. |  |
|  | 24b | A protocol was not prepared. |  |
|  | 24c | Nothing. |  |
| Support | 25 | This study was supported by Natural Science Foundation of Xinjiang Uygur Autonomous Region (No.2021D01C278). The funders had no role in study design, data collection and analysis, decision to publish, or preparation of the manuscript. | Funding statement in the online submission |
| Competing interests | 26 | We declare that we have no conflict of interest. | 16 |
| Availability of data, code and other materials | 27 | All of the data relevant in this study are publicly available with the attached information or online data base. | 17-19 |

*From:*  Page MJ, McKenzie JE, Bossuyt PM, Boutron I, Hoffmann TC, Mulrow CD, et al. The PRISMA 2020 statement: an updated guideline for reporting systematic reviews. BMJ 2021;372:n71. doi: 10.1136/bmj.n71

For more information, visit: <http://www.prisma-statement.org/>
