# Supplementary material for: Association of tumor necrosis factor-α-308G/A polymorphism with the risk of obstructive sleep apnea: A meta-analysis of 14 case-control studies
Source: PLoS One. 2023 Aug 18;18(8):e0290239. doi: 10.1371/journal.pone.0290239 (PMC10437904; doi:10.1371/journal.pone.0290239)
Supplement: S2 Table — (DOCX) [file pone.0290239.s002.docx]

**S2 Table. Search strategy**

January 8, 2022

The adopted appropriate combinations of search terms were as follows: “obstructive sleep apnea syndrome”, “OSAS”, “obstructive sleep apnea”, “OSA” and “tumor necrosis factor”, “TNF”, “tumor necrosis factor-a”, “TNF-α” and “polymorphism”, “gene”, “variant”, and “mutation”.

| #1 | (sleep apnea syndromes)[MeSH Terms] OR obstructive sleep apnea syndrome[Title/Abstract] OR OSAS [Title/Abstract] OR obstructive sleep apnea [Title/Abstract] OR OSA [Title/Abstract] |
| --- | --- |
| #2 | (tumor necrosis factor alpha)[MeSH Terms] OR tumor necrosis factor [Title/Abstract] OR TNF [Title/Abstract] OR tumor necrosis factor-a [Title/Abstract] OR TNF-α[Title/Abstract] |
| #3 | polymorphism [Title/Abstract] OR gene [Title/Abstract] OR variant [Title/Abstract] OR mutation [Title/Abstract] |
| #4 | #1 and #2 and #3 |
