# Supplementary figures and images for: Association of tumor necrosis factor-α-308G/A polymorphism with the risk of obstructive sleep apnea: A meta-analysis of 14 case-control studies
Source: PLoS One. 2023 Aug 18;18(8):e0290239. doi: 10.1371/journal.pone.0290239 (PMC10437904; doi:10.1371/journal.pone.0290239)

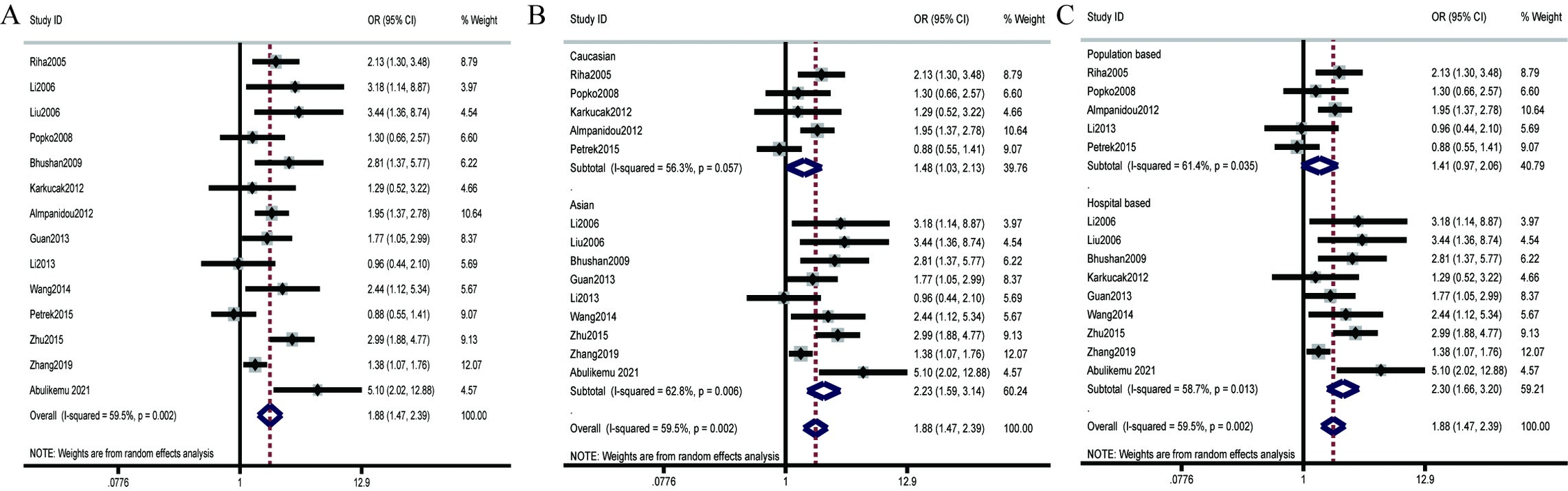

Supplement: S1 Fig — A: overall results; B: subgroup analysis by ethnicity; C: subgroup analysis by source of control. (TIF) [file pone.0290239.s003.tif]

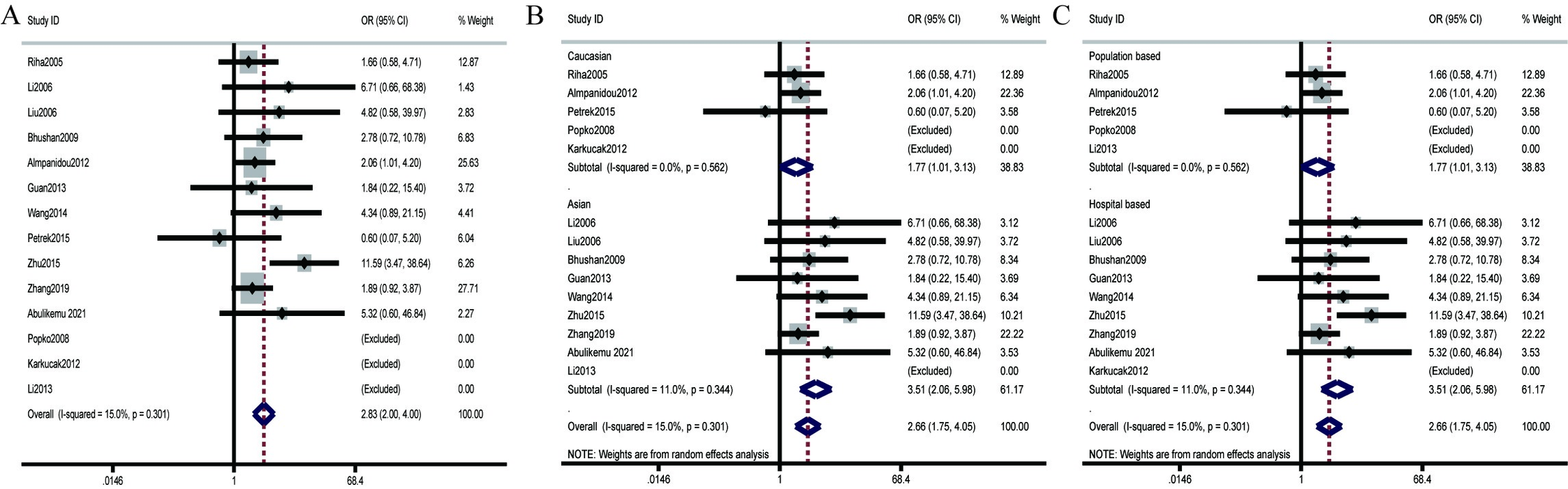

Supplement: S2 Fig — A: overall results; B: subgroup analysis by ethnicity; C: subgroup analysis by source of control. (TIF) [file pone.0290239.s004.tif]

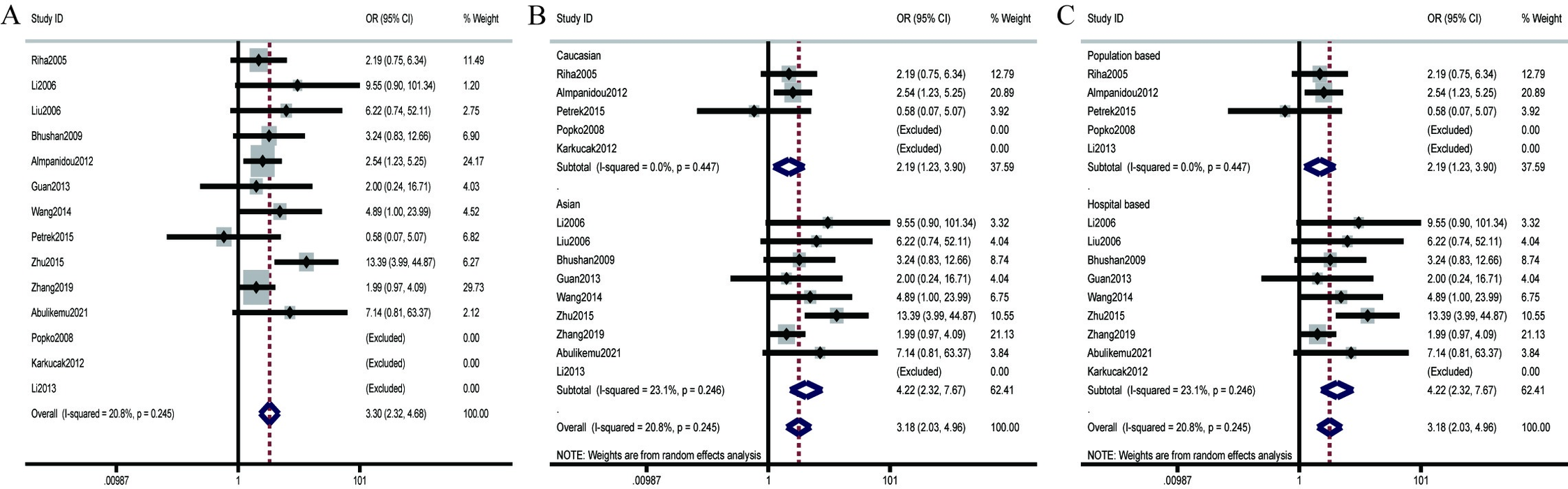

Supplement: S3 Fig — A: overall results; B: subgroup analysis by ethnicity; C: subgroup analysis by source of control. (TIF) [file pone.0290239.s005.tif]

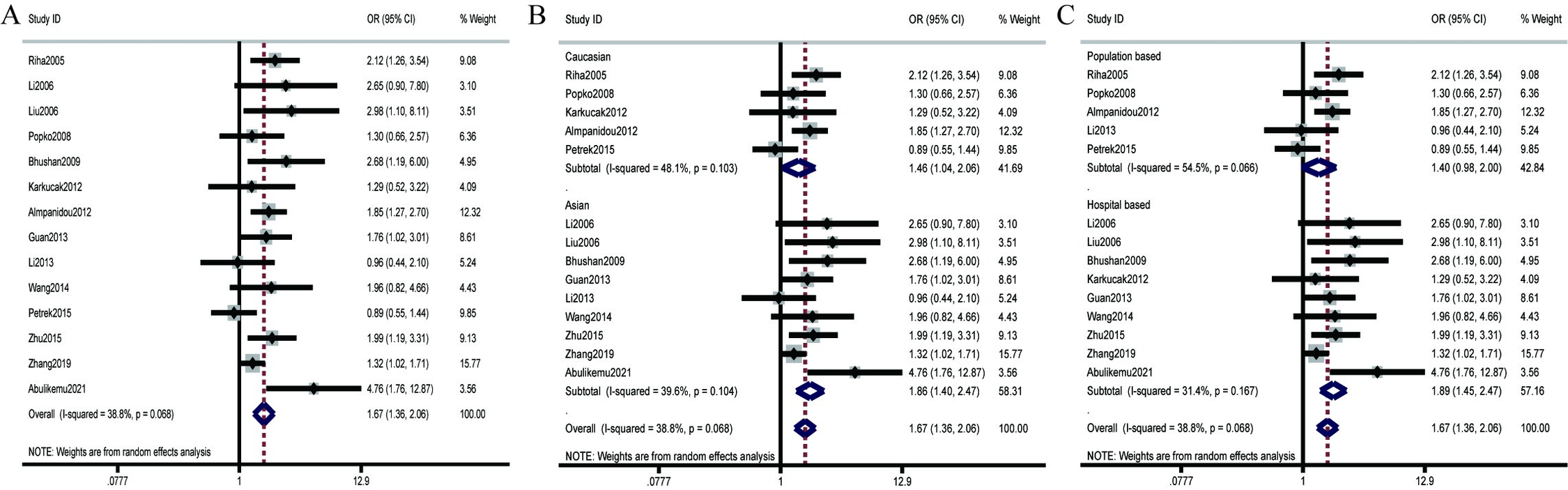

Supplement: S4 Fig — A: overall results; B: subgroup analysis by ethnicity; C: subgroup analysis by source of control. (TIF) [file pone.0290239.s006.tif]
