# Supplementary material for: Association of tumor necrosis factor-α-308G/A polymorphism with the risk of obstructive sleep apnea: A meta-analysis of 14 case-control studies
Source: PLoS One. 2023 Aug 18;18(8):e0290239. doi: 10.1371/journal.pone.0290239 (PMC10437904; doi:10.1371/journal.pone.0290239)
Supplement: Attachment — Submitted filename: PLOS ONE Reviewer TNFa.pdf [file pone.0290239.s007.pdf]

PLOS ONE
Association of Tumor Necrosis Factor-ɑ-308G/A polymorphism with the risk of obstructive sleep apnea: An updated meta-analysis of 14 case-control studies.
Reviewer

1. Is the manuscript technically sound, and do the data support the conclusions? Yes.
This manuscript is a meta-analysis of the association between Tumor Necrosis Factor-ɑ-308G/A polymorphism and obstructive sleep apnea. The statistical analysis of 14 case-control studies selected for this review was conducted rigorously and this meta-analysis is appropriately based on the Prisma checklist. The introduction of this manuscript reported previous meta-analyses in this area and explained that they included additional case-control studies.

The conclusions are based on the findings obtained through adequate statistical analysis. However, the discussion should include some limitations related to the genetic association studies and the fact that the studies included in this meta-analysis did not report the effect of AIH (apnea, hypoapnea index), oxygen saturation levels, arousals, comorbidities, sex, age, and other factors that could play an important role in the pathophysiology of the obstructive sleep apnea.

2. Has the statistical analysis been performed appropriately and rigorously? Yes.
The methodology is appropriate and rigorous. The inclusion and exclusion criteria are well formulated. The statistical analysis used in this meta-analysis included: Pearson's $\chi 2$ test performed to evaluate the Hardy–Weinberg equilibrium (HWE), Cochran's Q and the I2 statistics to evaluate heterogeneity, Z and OR test to evaluate genetic models, Harbord funnel plot and Peters test to identify publication bias. The results of these analyses are shown in tables and figures (forest plots, Harbord funnel plot) and show complete information about the association between Tumor Necrosis Factor-ɑ-308G/A polymorphism and obstructive sleep apnea reported in case-control studies.

3. Have the authors made all data underlying the findings in their manuscript fully available? Yes.
Data from each case-control study is described in the manuscript. Prisma checklist is supporting information. Complete and broad statistic results are

shown. The references included the articles selected for this meta-analysis and previous meta-analyses among others.

4. Is the manuscript presented in an intelligible fashion and written in standard English? Yes.
