# Supplementary material for: Association of tumor necrosis factor-α-308G/A polymorphism with the risk of obstructive sleep apnea: A meta-analysis of 14 case-control studies
Source: PLoS One. 2023 Aug 18;18(8):e0290239. doi: 10.1371/journal.pone.0290239 (PMC10437904; doi:10.1371/journal.pone.0290239)
Supplement: Attachment — Submitted filename: Response to Reviewers.docx [file pone.0290239.s008.docx]

Dear Editor and Reviewers,

Thank you very much for all your insightful comments and suggestions. They have been very helpful. We carefully modified the manuscript accordingly. All the revised portions of the manuscript are now marked in red (except in the clean version of the revised manuscript, also submitted). Please, see below for our responses (in blue) to all the comments/suggestions.

**Journal Requirements:**

1. Please ensure that your manuscript meets PLOS ONE's style requirements, including those for file naming. The PLOS ONE style templates can be found at

[https://journals.plos.org/plosone/s/file?id=wjVg/PLOSOne_formatting_sample_main_body.pdf](https://journals.plos.org/plosone/s/file?id=wjVg/PLOSOne_formatting_sample_main_body.pdf) and [https://journals.plos.org/plosone/s/file?id=ba62/PLOSOne_formatting_sample_title_authors_affiliations.pdf](https://journals.plos.org/plosone/s/file?id=ba62/PLOSOne_formatting_sample_title_authors_affiliations.pdf)

Response: Thank you for this comment. We have carefully revised the manuscript according to the PLOS ONE style templates.All the revised portions of the manuscript are now marked in red (except in the clean version of the revised manuscript, also submitted).

2. Please provide additional details regarding participant consent. In the ethics statement in the Methods and online submission information, please ensure that you have specified (1) whether consent was informed and (2) what type you obtained (for instance, written or verbal, and if verbal, how it was documented and witnessed). If your study included minors, state whether you obtained consent from parents or guardians. If the need for consent was waived by the ethics committee, please include this information.

If you are reporting a retrospective study of medical records or archived samples, please ensure that you have discussed whether all data were fully anonymized before you accessed them and/or whether the IRB or ethics committee waived the requirement for informed consent. If patients provided informed written consent to have data from their medical records used in research, please include this information.

Response: Thank you for this comment. Participant consent is not applicable since the present meta-analysis was based on published data.

3. Thank you for stating in your Funding Statement:

“This study was supported by Natural Science Foundation of Xinjiang Uygur Autonomous Region (No.2021D01C278) received by ZZL. The funders had no role in study design, data collection and analysis, decision to publish, or preparation of the manuscript.”

Please provide an amended statement that declares *all* the funding or sources of support (whether external or internal to your organization) received during this study, as detailed online in our guide for authors at [http://journals.plos.org/plosone/s/submit-now.](http://journals.plos.org/plosone/s/submit-now.)  Please also include the statement “There was no additional external funding received for this study.” in your updated Funding Statement.

Please include your amended Funding Statement within your cover letter. We will change the online submission form on your behalf.

Response: Thank you for this comment. The amended Funding Statement marked in red has been within our cover letter accordingly.

4. In your Data Availability statement, you have not specified where the minimal data set underlying the results described in your manuscript can be found. PLOS defines a study's minimal data set as the underlying data used to reach the conclusions drawn in the manuscript and any additional data required to replicate the reported study findings in their entirety. All PLOS journals require that the minimal data set be made fully available. For more information about our data policy, please see [http://journals.plos.org/plosone/s/data-availability.](http://journals.plos.org/plosone/s/data-availability.)

"Upon re-submitting your revised manuscript, please upload your study’s minimal underlying data set as either Supporting Information files or to a stable, public repository and include the relevant URLs, DOIs, or accession numbers within your revised cover letter. For a list of acceptable repositories, please see [http://journals.plos.org/plosone/s/data-availability#loc-recommended-repositories.](http://journals.plos.org/plosone/s/data-availability#loc-recommended-repositories.) Any potentially identifying patient information must be fully anonymized.

Important: If there are ethical or legal restrictions to sharing your data publicly, please explain these restrictions in detail. Please see our guidelines for more information on what we consider unacceptable restrictions to publicly sharing data: [http://journals.plos.org/plosone/s/data-availability#loc-unacceptable-data-access-restrictions.](http://journals.plos.org/plosone/s/data-availability#loc-unacceptable-data-access-restrictions.) Note that it is not acceptable for the authors to be the sole named individuals responsible for ensuring data access.

We will update your Data Availability statement to reflect the information you provide in your cover letter.

Response: Thank you for this comment. Since the present meta-analysis was based on published data, all relevant data are within the manuscript and its Supporting information files.The updated Data Availability statement marked in red has been within our cover letter accordingly.

5**.** Please include captions for your Supporting Information files at the end of your manuscript, and update any in-text citations to match accordingly. Please see our Supporting Information guidelines for more information: [http://journals.plos.org/plosone/s/supporting-information.](http://journals.plos.org/plosone/s/supporting-information.)

Response: Thank you for this comment. We have listed Supporting Information captions at the end of the manuscript in a section titled “Supporting information”.

**Reviewer #1:**

This manuscript is a meta-analysis of the association between Tumor Necrosis Factor-α-308G/A polymorphism and obstructive sleep apnea. The statistical analysis of 14 case-control studies selected for this review was conducted rigorously and this meta-analysis is appropriately based on the Prisma checklist. The introduction of this manuscript reported previous meta-analyses in this area and explained that they included additional case-control studies.

The conclusions are based on the findings obtained through adequate statistical analysis. However, the discussion should include some limitations related to the genetic association studies and the fact that the studies included in this meta-analysis did not report the effect of AIH (apnea, hypoapnea index), oxygen saturation levels, arousals, comorbidities, sex, age, and other factors that could play an important role in the pathophysiology of the obstructive sleep apnea.

The methodology is appropriate and rigorous. The inclusion and exclusion criteria are well formulated. The statistical analysis used in this meta-analysis included: Pearson’s χ2 test performed to evaluate the Hardy–Weinberg equilibrium (HWE), Cochran’s Q and the I2 statistics to evaluate heterogeneity, Z and OR test to evaluate genetic models, Harbord funnel plot and Peters test to identify publication bias. The results of these analyses are shown in tables and figures (forest plots, Harbord funnel plot) and show complete information about the association between Tumor Necrosis Factor-α-308G/A polymorphism and obstructive sleep apnea reported in case-control studies.

Data from each case-control study is described in the manuscript. Prisma checklist is supporting information. Complete and broad statistic results are shown. The references included the articles selected for this meta-analysis and previous meta-analyses among others.

Response: Thank you for your comment.According to your suggestion, we have revised the second limitation in discussion:The etiology of OSA is likely multifactorial, however, sex, age, body mass index, life style, AHI, comorbidities, other gene polymorphisms and other factors that may play important roles in the pathophysiology of OSA were not available in most eligible studies. Therefore gene–gene or gene–environment interactions were not evaluated in the present meta-analysis.Thus, the second limitation may be unadjusted pooled estimates by potential confounding factors.

Thank you and best regards.

Yours sincerely,

Qiao ling Yao

**Corresponding author:Qiao-ling Yao,PhD**

Department of Physiology, School of Basic Medical Sciences, Xinjiang Medical University, Urumqi, Xinjiang, China.

Email: 49165627@ qq.com

Mobile Number:15022917911
