# Supplementary material for: Association of tumor necrosis factor-α-308G/A polymorphism with the risk of obstructive sleep apnea: A meta-analysis of 14 case-control studies
Source: PLoS One. 2023 Aug 18;18(8):e0290239. doi: 10.1371/journal.pone.0290239 (PMC10437904; doi:10.1371/journal.pone.0290239)
Supplement: Attachment — Submitted filename: Response to Reviewers.docx [file pone.0290239.s009.docx]

Dear Editor and Reviewers,

Thank you very much for all your insightful comments and suggestions. They have been very helpful. We carefully modified the manuscript accordingly. All the revised portions of the manuscript are now marked in red (except in the clean version of the revised manuscript, also submitted). Please, see below for our responses (in blue) to all the comments/suggestions.

**Journal Requirements:**

1.Please review your reference list to ensure that it is complete and correct. If you have cited papers that have been retracted, please include the rationale for doing so in the manuscript text, or remove these references and replace them with relevant current references. Any changes to the reference list should be mentioned in the rebuttal letter that accompanies your revised manuscript. If you need to cite a retracted article, indicate the article’s retracted status in the References list and also include a citation and full reference for the retraction notice.
Response: Thank you for this comment. We have carefully reviewed reference list and ensureed that it is complete and correct.

**Additional Editor Comments:**

Abstract
Line # 31
OSA was used for the first time in this section; kindly spell it out.
Response: Thank you for this comment. We have spelled it out.

Line # 35
Please replace the term ‘used” with “accessed”
As the authors describe how to estimate association in the current metanalysis, it should be "pooled odds ratio or summary odds ratio."
Response: Thank you for this comment. We have replaced the term “used” , “pooled odds ratio” with “accessed”and “pooled odds ratio”,respectively.

Line # 40-42; Line # 44-45; Line # 48-49
Please provide number of studies for the respective effect estimates.
Response: Thank you for this comment. We have provided number of studies for the respective effect estimates.

Line # 49-50
“Substantial between-study heterogeneity” is preferable.
“ p value” of the I2 test should be deleted.
Response: Thank you for this comment. We have replaced the term “statistically significant heterogeneity” with “Substantial between-study heterogeneity”. “ p value” of the I^2^ test has be deleted.

Line # 51
Better to describe as “Source of control participants”.
Response: Thank you for this comment. We have replaced the term “source of control” with “Source of control participants”.

What sensitivity analysis is it for? Please provide specifically. Sensitivity analysis for "source of control participants" was reported in the text.

Response: Thank you for this comment. Sensitivity analysis refers to the method of testing the robustness of the results obtained under certain assumptions, and observing whether the pooled results have changed before and after changing some important factors, so as to judge the robustness of the results of meta-analysis. In this study,we conducted a sensitivity analysis by removing individual studies one by one to observe the difference between the combined and total effects of the remaining studies, and examined the impact of individual studies. As described in line199-200: as presented in Fig 3B, results of sensitivity analysis showed no significant effect on the pooled ORs by any single study, further confirming the reliability and stability of the results.

In the abstract, I feel a lack of recommendation in the conclusions. Based on the study limitations that the authors identified during the current research, please suggest future well-designed case control studies, etc.
Response: Thank you for this comment. Recommendation has been added in the conclusions:However, large scale,multi-center and well-designed case-control studies are also needed in the future.

TEXT
Line # 87
Please omit the word "an updated". Publications from the past were not done by this team.

Response: Thank you for this comment. The word "an updated" has been omitted.

Search Strategy
Please provide “search strategy for PubMed and Embase in supplementary so that the audiences can replicate.
Response: Thank you for this comment. Search strategy has been provided in S2 table.

Line #101
Criteria # 1 is a doubtful. Did you select studies that showed only susceptible to OSA, but not protective. In earlier part, the authors claimed that “the results (from single studies) remained controversial and ambiguous”.
The authors should have selected studies that assessed association between TNF-α-308G/A polymorphisms and OSA (regardless of direction of association)
Response: Thank you for this comment.The word " the susceptibility of " has been omitted.

Line # 111
“The definition of OSA was as described in the primary studies” is preferable.
Response: Thank you for this comment. We have replaced the term “the definition of OSA used in each eligible study was accepted” with “The definition of OSA was as described in the primary studies”.

Line # 117
HWE should have been spelled out as the authors didn't use an abbreviation.
Response: Thank you for this comment. We have spelled it out.

Line # 120
"Missing" is preferable than "ambiguous" as a description.
Response: Thank you for this comment. We have replaced the term “ambiguous” with “Missing”.

Line # 124
To the best of my understanding, the NOS checklist is a 9-star tool, with 9 being the highest possible star/score. It is impossible to score more than 9.

Response: Thank you for this comment. We have changed it to 9.

Line # 117
The abbreviation HWE may be used at this juncture (please refer to my comments on line #117).

Response: Thank you for this comment. The abbreviation HWE has been used.

Line # 155; 172-175; 178 Please provide number of studies for the respective effect estimates.
Response: Thank you for this comment. We have provided number of studies for the respective effect estimates.

Line #188
This should come at the top of this paragraph.

Response: Thank you for this comment. It has been at the top of this paragraph

Line # 198
For the dominant model and heterozygous models, please provide p values.

Response: Thank you for this comment. P values for the dominant model and heterozygous models have been provided.

What about the remaining models?

Response: Thank you for this comment. Meta-regression was conducted to explore potential source of between-study heterogeneity, therefore, we only conducted meta-regression analysis on allele,dominant and heterozygous models.

Please remove p value for I2 test.
Response: Thank you for this comment. We did not remove the *P* value(*P=*0.036) because it is the *P* value of meta regression.

Table #2
Please remove the last column with I2 test p value.
Response: Thank you for this comment. We have removed the last column with I2 test p value.

Discussion
This section requires more efforts.
As this is a meta-analysis, citations to individual studies should be reduced/shortened.
Please briefly describe how the current study and previous reviews in this field are compared and contrasted.

Response: Thank you for this comment. We have carefully modified the discussion.

Please use a subheading of the “study limitations” for clarity

Response: Thank you for this comment. Subheading of the “study limitations” has been added.

To add a recommendation as a concluding remark. Please note my recommendation for this section of the abstract.
Response: Thank you for this comment.Recommendation has been added: however, large scale,multi-center and well-designed case-control studies are also needed in the future. Meanwhile, it is also of great significance to explore the mechanism of TNF-α-308 G/A polymorphism involved in OSA pathogenesis.

**Reviewers' comments:**
Review Comments to the Author

Reviewer #2: The manuscript was well written. Introduction clearly and adequately stated the background of the current issue of the obstructive sleep apnea. Nevertheless, there are some suggestions as follow.

Results:
Table 1- Basic Characteristics of included studies
In this table, suggest to provide reference number in addition to author names.
Response: Thank you for this comment. Reference number has been added in table1.

In figures, it would be great if authors could provide the forest plots of effect estimates for each genetic models as a supplemenatary file.

Response: Thank you for this comment. The forest plots of effect estimates for each genetic models have been provided as supplemenatary figures.

Thank you and best regards.

Yours sincerely,

Qiao ling Yao

**Corresponding author:Qiao-ling Yao,PhD**

Department of Physiology, School of Basic Medical Sciences, Xinjiang Medical University, Urumqi, Xinjiang, China.

Email: 49165627@ qq.com

Mobile Number:15022917911
