# Supplementary material for: Association of tumor necrosis factor-α-308G/A polymorphism with the risk of obstructive sleep apnea: A meta-analysis of 14 case-control studies
Source: PLoS One. 2023 Aug 18;18(8):e0290239. doi: 10.1371/journal.pone.0290239 (PMC10437904; doi:10.1371/journal.pone.0290239)
Supplement: Attachment — Submitted filename: Response to Reviewers.docx [file pone.0290239.s010.docx]

Dear Editor and Reviewers,

Thank you very much for all your insightful comments and suggestions. They have been very helpful. We carefully modified the manuscript accordingly. All the revised portions of the manuscript are now marked in red (except in the clean version of the revised manuscript, also submitted). Please, see below for our responses (in blue) to all the comments/suggestions.

**Journal Requirements:**

1. Please review your reference list to ensure that it is complete and correct. If you have cited papers that have been retracted, please include the rationale for doing so in the manuscript text, or remove these references and replace them with relevant current references. Any changes to the reference list should be mentioned in the rebuttal letter that accompanies your revised manuscript. If you need to cite a retracted article, indicate the article’s retracted status in the References list and also include a citation and full reference for the retraction notice.
Response: Thank you for this comment. We have carefully reviewed reference list and ensureed that it is complete and correct.

**Additional Editor Comments:**

Title
Regarding my background comments, the phrase "an updated" has to be removed from the title.
Previous publications were not done by this team.

Response: Thank you for this comment. The phrase "an updated" has been removed from the title.

Abstract
Line #32………. “Obstructive” should not be capitalized

Response: Thank you for this comment. “Obstructive” has been capitalized.

Line #41……… Please include the names of genetic models that were created for overall population. I believe that overall population was assessed using five different genetic models such as allele. "Dominant, recessive, homozygous, and heterozygous models"

Response: Thank you for this comment. The names of genetic models that were created for overall population has been included.

Line # 47, 49……….. To spell out HB and PB

Response: Thank you for this comment. HB and PB have been spelled out.

Line # 50…………. Name the genetic models for AA in comparison to GG, AG/GG

Response: Thank you for this comment. We have named the genetic models for AA in comparison to GG, AG/GG.

Line # 53…………. The statement "a leave-one-out meta-analysis" should be applied.

Response: Thank you for this comment. The statement "a leave-one-out meta-analysis" has been applied.

Line # 56……… Take out the word “also”

Response: Thank you for this comment. The word “also” has been taken out.

**TEXT**
Line # 63……….. replacing "published studies reported" for "increasing evidence has demonstrated"

Response: Thank you for this comment. We have replaced "published studies reported" for "increasing evidence has demonstrated".

Line # 68/ 69………. Please rephrase this sentence. The word "thus" was used twice

Response: Thank you for this comment. We have.rephrased this sentence.

Line # 72………. The wording "by Szily and coworkers or By Szily and associates" should have been used.

Response: Thank you for this comment. The wording "by Szily and coworkers" has been used.

Line # 72………. Replace the word "strong evidence" with lighter tone, like "published studies."

Response: Thank you for this comment. We have replaced "strong evidence" for "published studies".

Line # 78, 79, 80…… The authors could combine these two sentences into one and then cite [13] afterward.

Response: Thank you for this comment. We have combined these two sentences into one and then cited [13] afterward.

Line # 90………. “Hence” is a preferrable term.

Response: Thank you for this comment. We have replaced "Herein" for " Hence ".

Line # 92………. “Source of the control participants”

Response: Thank you for this comment. We have replaced "control source " for " source of the control participants".

Line # 114……… Please, remove “additionally”.

Response: Thank you for this comment. We have removed “additionally”.

Line # 133……… Please keep "homozygous" and "heterozygous" while removing "codominant."

Response: Thank you for this comment. We have removed “codominant”.

Line # 138……The word .”Otherwise” is preferrable to “By contrast”..

Response: Thank you for this comment. We have replaced "By contrast" for "Otherwise ".

Line # 141……. So as to differentiate it from statistical models (Random. Fixed models), it needs to be mentioned as "for all genetic models".

Response: Thank you for this comment. We have replaced "for all models " for " for all genetic models ".

Line # 141-142………… Are you referring to "a leave-one-out metanalysis" for the stability of the results.

Response: Thank you for this comment.Yes.

Line # 146……… Literature search and characteristics of included studies.
Please separate the subheadings into two sections, such as "Selection of Studies" and "Characteristics of the Included Studies."

Response: Thank you for this comment. We have separated the subheadings into "Selection of Studies" and "Characteristics of the Included Studies."

Line # 157……… This sentence should be placed at the beginning of paragraph, “Selection of studies”.

Response: Thank you for this comment. This sentence “The process of study selection was presented in Fig 1.” has been placed at the beginning of paragraph “Selection of studies”.

Line # 160, 165 and 166……… It is better to use "eligible". (Please follow this suggestion wherever it is applicable.)

Response: Thank you for this comment. We have replaced "enrolled " for " eligible ".

Line # 162……… Source of the control participants?

Response: Thank you for this comment. "Source of the control participants " has been used.

Line # 167-168……… Please rephrase this sentence.

Response: Thank you for this comment.We have rephrased this sentence.

Table 1…….. Footnotes are incomplete. HB? PB?
Last column…….. Quality score?

Response: Thank you for this comment.” PB,population-based; HB,hospital-based ” have been existed in footnotes. We have replaced " score " for " Quality score " in last column.

Line # 175……… Please add the names of the genetic models for the audiences' convenience. Apply this recommendation to Table 2 as well by including a new column for the genetic model's name.

Response: Thank you for this comment. We have added the names of the genetic models and included a new column for the genetic model's name in Table 2.

Line # 204-205………Please clearly indicate that the results are "results of a leave-one-out metanalysis" (also see my comments on Lines 53 and 141–142).

Response: Thank you for this comment. We have replaced "sensitivity analysis " for " eligible " leave-one-out meta-analysis".

**Study limitations**
Line # 273…………….. Stability is an appropriate term. What about the reliability? Which analysis/results captured the reliability?

Response: Thank you for this comment. “Reliability” has been removed.

Line # 273…………….. Language bias or information bias?

Line # 275-283……………..Please rephrase the sentences and shorten it.
First, language bias/informational bias? Second, the confounding bias created by……. Third, not all studies conform to the HWE. So, what type of bias? Fourth, there could be additional factors that have not been included in this review.

Response: Thank you for this comment. We have modified it as follows: First, language bias resulted from limition to publication in English and Chinese. Second, the confounding bias created by unavailable factors in most eligible studies like sex, age, body mass index, life style, AHI, comorbidities, other gene polymorphisms that may play important roles in the pathophysiology of OSA. Third, sampling bias resulted from enrolling a study unconforming to HWE law. Fourth, there could be additional factors that have not been included in this review.

**Acknowledgements**
Are you thanking your research team members or the participants and researchers in the studies included in your review? Please clarify.

Response: Thank you for this comment. We have modified it as follows: We appreciate the contribution of participants and researchers in the studies included in this review.

Thank you and best regards.

Yours sincerely,

Qiao ling Yao

**Corresponding author:Qiao-ling Yao,PhD**

Department of Physiology, School of Basic Medical Sciences, Xinjiang Medical University, Urumqi, Xinjiang, China.

Email: 49165627@ qq.com

Mobile Number:15022917911
